# Systemic Embolization from an Unusual Intracardiac Mass in the Left Ventricular Outflow Tract

**DOI:** 10.1155/2017/4513623

**Published:** 2017-06-01

**Authors:** Kelechukwu U. Okoro, Timothy R. Larsen, John C. Lystash

**Affiliations:** Virginia Tech Carilion School of Medicine and Carilion Medical Center, Roanoke, VA, USA

## Abstract

Endocarditis can affect any endocardial surface; in the vast majority of cases, the cardiac valves are involved. It is exceedingly rare to develop infective endocarditis on the endocardium of the left ventricular outflow tract due to the high velocity of blood that traverses this area. Herein, we present a rare case of left ventricular outflow tract endocarditis that likely occurred secondary to damage to the aortic valve leaflets (from healed prior aortic valve endocarditis) causing a high velocity aortic valve regurgitant jet that impinged upon the interventricular septum which damaged the endocardium and resulted in a fibrotic “jet lesion.” This fibrous jet lesion served as a nidus for bacterial proliferation and vegetation formation. The high shear stress (due to high blood flow velocity through the left ventricular outflow tract) likely promoted the multiple embolic events observed in this case. Our patient was successfully treated with aortic valve replacement, vegetation resection, and antibiotics.

## 1. Introduction

The differential diagnosis for intracardiac masses includes thrombus, vegetation, benign primary cardiac tumors, malignant primary cardiac tumors, and metastatic secondary cardiac tumors [1, 2]. Diagnosis is usually suggested by a combination of history, physical examination, and imaging. Transthoracic echocardiography (TTE) often provides valuable information when diagnosing cardiac masses. Transesophageal echocardiography (TEE) is the imaging modality of choice due to the proximity of the interface between the probe and the heart producing a high sensitivity and specificity [1–5]. TEE can also better identify complications related to masses including abscesses and fistulae [3, 4]. Other potentially useful imaging modalities include cardiac magnetic resonance, cardiac computed tomography, and cardiac positron emission topography [2, 3]. Once appropriate imaging and diagnosis have been obtained, an appropriate plan of action can be undertaken; for example, cardiac surgery may be indicated in select cases of infective endocarditis. Herein, we present an unusual case of infective endocarditis arising from the left ventricular outflow tract.

## 2. Case Report

A 33-year-old female presented to the emergency room complaining of painful lesions on her right upper extremity that began approximately two days prior to presentation. She also noted generalized malaise, chills, subjective fever, nausea, vomiting, abdominal pain, and dyspnea on exertion. She became short of breath with minimal activity. Past medical history was significant for hepatitis C, polysubstance abuse, and nicotine dependence. Approximately six months prior to current illness, she was hospitalized and treated for staphylococcal left knee septic arthritis.

**Figure 1:**
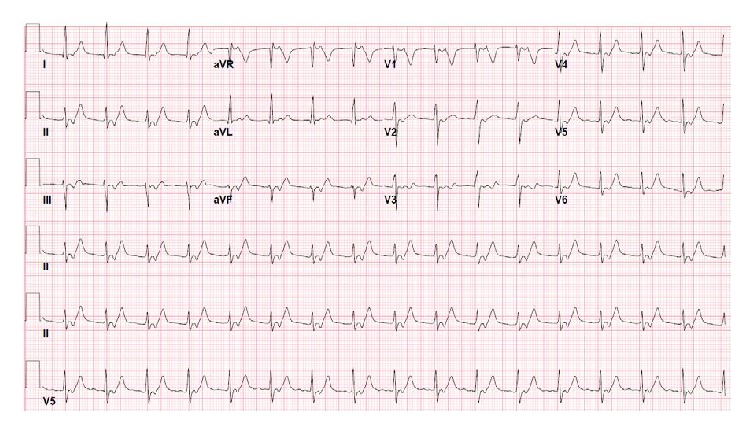
Initial EKG demonstrating accelerated junctional rhythm at 99 beats/minute with retrograde P wave conduction, QS duration 92 ms, and corrected QT interval 469 ms.

Initial vital signs were BP 144/71 mmHg, pulse 107 beats/min, temperature 98.1°F (36.7°C), respiratory rate 20 breaths/min, and oxygen saturation 99% breathing ambient air. Physical examination demonstrated petechiae and ecchymosis on all four extremities along with several tender erythematous nodules. She also had jugular venous distention to the angle of the mandible. There were bibasilar rales on pulmonary examination and a 2/6 intensity holodiastolic murmur was present at the 2nd right interspace on cardiac examination. There was mild bilateral lower extremity pitting edema. The remainder of the physical exam was unremarkable. Laboratory data demonstrated serum sodium 131 mEq/L, potassium 3.0 mEq/L, chloride 97 mEq/L, bicarbonate 24 mEq/L, BUN 21 mg/dl, creatinine 1.01 mg/dl, and glucose of 110 mg/dL. Cardiac troponin was undetectable. White blood cell count was 9.3 k/*μ*l, hemoglobin 14.3 g/dl, hematocrit 41.5%, and platelet count 99 k/*μ*l. Blood cultures grew Methicillin Sensitive* Staphylococcus aureus* (MSSA). Electrocardiogram demonstrated accelerated junctional rhythm at 99 beats/minute with retrograde P waves (Figure 1).

**Figure 2:**
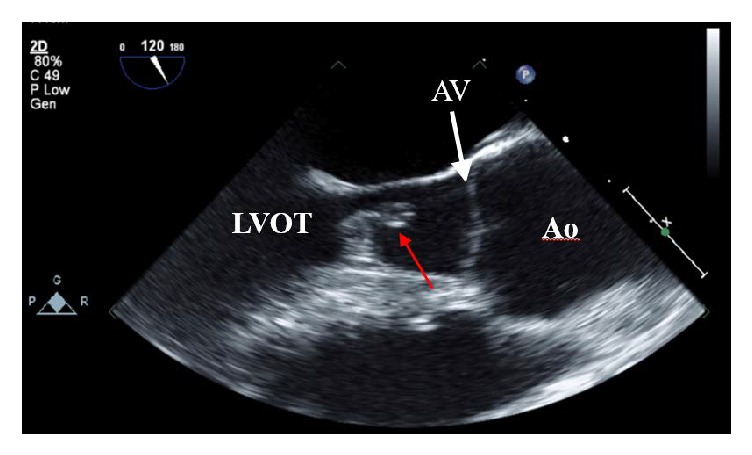
Transesophageal echocardiogram mid-esophageal position demonstrating a 1.3 by 1.0 cm mass (red arrow) attached to the interventricular septum in the left ventricular outflow tract during diastole. LVOT = left ventricular outflow tract, Ao = aorta, and AV = aortic valve.

**Figure 3:**
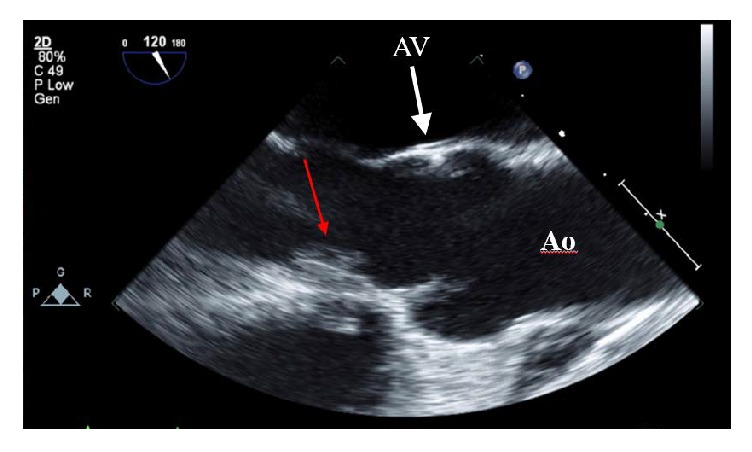
Transesophageal echocardiogram mid-esophageal position. The mass moves out of view during systole; red arrow indicates location of mass attachment to the left ventricular outflow tract. Ao = aorta and AV = aortic valve.

**Figure 4:**
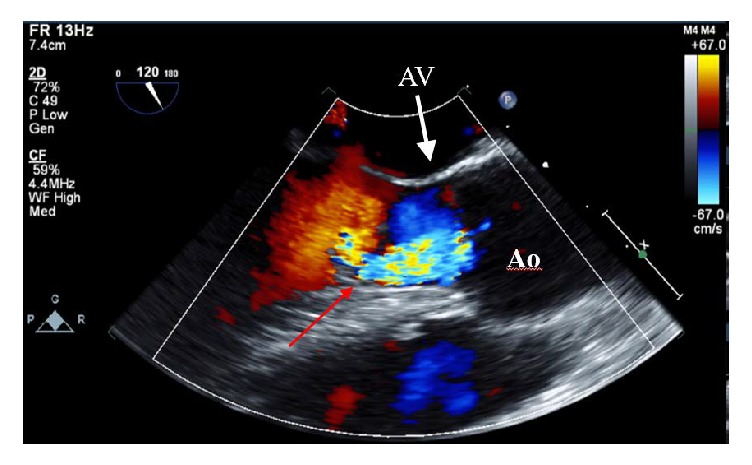
Transesophageal echocardiogram with color flow Doppler showing the aortic regurgitant jet (blue color) contacting the septal wall of the left ventricular outflow tract at the attachment site of the mass (red arrow). Ao = aorta and AV = aortic valve.

Transthoracic echocardiogram revealed normal LV chamber dimension, wall motion, and left ventricular ejection fraction estimated at 60–65%. The aortic valve was not clearly visualized but aortic regurgitation was noted with continuous wave and color flow Doppler. Transesophageal echocardiogram revealed severe aortic regurgitation; pressure half-time of the regurgitant jet was 320 ms. A large mobile mass measuring 1.3 cm by 1.0 cm was attached to the septal wall of the left ventricular outflow tract (LVOT) (Figures 2 and 3). The mass was attached at the site of a jet lesion where the aortic valve regurgitant jet contacted the LVOT wall (Figure 4).

She was treated with intravenous antibiotics. Due to the size and location of the vegetation, she was referred for aortic valve replacement and resection of the LVOT vegetation. Pathologic analysis of the native aortic valve revealed a 0.6 × 0.4 cm ovoid perforation of the noncoronary leaflet likely due to prior endocarditis with acute vegetation on the septal surface of the LVOT under the right coronary leaflet. The patient did well postoperatively and was treated adequately with a six-week course of intravenous antibiotics.

## 3. Discussion

It is likely that the MSSA left knee septic arthritis she was treated from six months ago was due to septic embolization or hematologous spread from unrecognized subacute aortic valve endocarditis, which also caused the aortic valve noncoronary cusp perforation. The antibiotic therapy for septic arthritis given at that time also partially treated the endocarditis. The perforation in the aortic leaflet produced a regurgitant jet directed towards the septal portion of the LVOT creating a jet lesion on the septum. A jet lesion occurs due to a high velocity stream of blood flow through an ostium or fenestration either secondary to a stenosed or regurgitant valve or in our case perforation of the valve leaflet [6]. The area of endocardium that is impinged upon by the regurgitant jet is damaged due to shear forces and friction from the high velocity of the jet. The sites of endothelial injury are highly thrombophilic promoting deposition and aggregation of platelets and fibrin [7]. The conglomeration of fibrin, platelets, and damaged endothelium produces a rich nidus for bacterial proliferation and is referred to as a MacCallum plaque [6, 7]. The regurgitant jet may also facilitate retrograde dissemination of infection [8].

Once a plaque or jet lesion distant from the original site of infection becomes seeded with bacteria it is referred to as a satellite lesion [6]. Satellite lesions with aortic regurgitation often occur on the anterior leaflet of the mitral valve or on the mitral chordae tendinae most commonly when the noncoronary cusp or the adjacent commissure of the aortic valve is involved [6, 8]. Damage to the mitral valve can include weakening of the leaflet substance with ulceration or aneurysm formation to perforation of the leaflet and ruptured chordae tendinae [8]. Satellite lesions within the LVOT itself are uncommon; likely the high velocity flow during ventricular systole prevents attachment of bacteria in this region of the heart. Vegetations typically occur on the aortic side of the aortic valve or the atrial side of the mitral valve where flow velocities are lowest [1]. We believe that the jet lesion created by the aortic valve regurgitant jet was necessary for this unusual vegetation to form.

Our patient underwent urgent surgery due to concern for recurrent embolization considering the size and mobility of the vegetation combined with high shear stress from high blood flow velocity in the LVOT. Additionally, she had acute heart failure due to severe aortic valve insufficiency. The objective of surgery is to remove infected tissue, foreign material, and hardware; clear and debride paravalvular infection and cavities; restore cardiac integrity and valve function; and remove threatening sources of emboli [3]. It is imperative that she abstains from recreational intravenous drug use in the future to avoid a repeat episode of infective endocarditis.

## 4. Conclusion

Left ventricular outflow tract endocarditis is a rare occurrence that occurs secondary to damage of the epicardium due to turbulent blood flow.
